# Number of motile spermatozoa inseminated and pregnancy outcomes in intrauterine insemination

**DOI:** 10.1186/s40738-019-0062-z

**Published:** 2019-09-02

**Authors:** Palma G. Gubert, Jessica Pudwell, Dean Van Vugt, Robert L. Reid, Maria P. Velez

**Affiliations:** Department of Obstetrics and Gynaecology, Division of Reproductive Endocrinology and Infertility, Queen’s University, Kingston Health Sciences Centre, 76 Stuart Street, Victory 4, Kingston, ON K7L 2V7 Canada

**Keywords:** Assisted reproduction, Intrauterine insemination, Pregnancy rates

## Abstract

**Purpose:**

To determine whether age modifies the effect of the number of motile spermatozoa inseminated (NMSI) as a predictor of success in Intrauterine Insemination (IUI).

**Methods:**

This retrospective cohort study included all patients who underwent IUI at an academic infertility center between October 2004 and June 2018. The primary outcome was clinical pregnancy (CP; a gestational sac and fetal heartbeat on ultrasound). Results were analyzed by patient factors including age, NMSI, duration of infertility, and cause of infertility, along with treatment factors such as number of follicles and ovulation induction protocol. Factors associated with the odds of achieving a clinical pregnancy were analyzed using binary logistic generalized estimating equations to control for clustering effects by couple. Female age was categorized as <35 years vs. ≥35 years.

**Results:**

Seven hundred thirty-seven couples that underwent 2062 IUI cycles for heterogeneous indications were included. The overall CP rate was 15.1% per cycle, and the cumulative CP rate per couple was 35.9%. For females < 35 years, the odds of CP per cycle were reduced for NMSI categories (× 10^6^) of < 5.0 vs. ≥10.0 (OR = 0.49; 95% CI 0.29–0.83); the odds of CP per cycle did not differ for NMSI 5.0–9.9 vs. ≥10.0 (OR = 0.66; 0.37–1.18). For those ≥35 years, no difference was seen in the odds of CP per cycle for NMSI categories < 5.0 vs. ≥10.0 (OR = 1.55; 95% CI 0.72–3.31) or 5.0–9.9 vs. ≥10.0 (OR = 1.04; 95% CI 0.48–2.27).

**Conclusions:**

These results suggest that the NMSI can be used as a predictor of success in IUI in couples with women who are < 35 years of age; these patients should be counselled about their lower pregnancy rates when the NMSI is < 5.0 × 10^6^. In patients ≥35 years, the NMSI does not appear to be a useful predictor of success. Further studies with larger sample size should be conducted.

## Background

Intrauterine insemination (IUI) is a cost-effective strategy and first line approach for the treatment of couples with unexplained and mild male infertility [1–3]. It involves the insertion of a high number of washed spermatozoa directly into the uterus at the time of ovulation to increase the chance of a pregnancy. IUI is often combined with ovarian stimulation (OS) to increase the number of eggs ovulating in a given cycle. Certain patient-related factors may indicate a poor chance of success with IUI, such as tubal disease or severe male factor infertility; such couples should be advised to proceed directly to other assisted reproductive technologies (ARTs), such as in vitro fertilization (IVF) and intracytoplasmic sperm injection (ICSI) when indicated.

Many factors have been considered for their potential role as both predictors and optimizers of the success of IUI, including type and duration of infertility, number of mature follicles, endometrial thickness, and various seminal parameters [4]. The sperm parameters most frequently examined in relation to pregnancy rates are (i) number of motile spermatozoa inseminated (NMSI); (ii) sperm morphology using strict criteria; (iii) total motile sperm count (TMSC) in the native sperm sample; and (iv) total motility in the native sperm sample [5]. Current evidence does not allow to define clear lower cut-off levels of pre- or postwash sperm parameters below which IUI should not be performed [1]. A recent systematic review concluded that a TMSC > 1 million and a morphology > 4% are of possible prognostic value, in such a case that below these cut-off levels IUI should be withheld, however the quality of evidence was low [5]. In 2004, a meta-analysis of 16 studies assessing NMSI and IUI outcomes, concluded that at cut-off levels between 0.8 and 5 million, the specificity of the NMSI, defined as the ability to predict failure to become pregnant, was as high as 100%; and the sensitivity of the test, defined as the ability to predict pregnancy, was limited [6]. Subsequent studies have been conducted [7, 8], however there is not yet a consensus on a minimum recommended NMSI threshold, below which, IUI is unlikely to result in a pregnancy.

Such NMSI thresholds are typically reported for female patients of all ages pursuing IUI, up to a maximum age [5, 7–10]. Female age is known to be an independent predictor of IUI success [11] but the effect of NMSI on pregnancy rates according to female age has been less studied [12, 13]. The purpose of this study was thus to determine whether age modifies the effect of NMSI as a predictor of success in IUI.

## Methods

This was a retrospective cohort study comprising patients that underwent IUI at the Fertility Clinic at the Kingston General Hospital in Kingston, Ontario, Canada between October 2004 and June 2018.

Before entry into the IUI program, patients were investigated to determine the cause of infertility, with investigations conducted as necessary to elicit etiology. Female patients had tubal patency confirmed by hysterosalpingogram, and men had a semen analysis. Causes of infertility were grouped into male factor, female factor (ovulatory dysfunction, mild to moderate endometriosis, and diminished ovarian reserve), combined male and female factors, and unexplained infertility.

The primary outcome was clinical pregnancy (CP), defined by a gestational sac and fetal heartbeat on ultrasound. The secondary outcomes were β-HCG positive pregnancy and live birth. Additional information collected from the chart review included duration of infertility, number of mature follicles, ovulation induction protocol, and live birth outcomes. Female age was categorized as <35 years vs. ≥35 years. Charts were excluded if the primary outcome (CP) or the primary exposure (NMSI) were missing.

### Ovulation induction protocol and monitoring

Most patients underwent OS ovarian stimulation with gonadotropins, while a minor proportion received clomiphene or letrozole, or a combination of clomiphene or letrozole and gonadotropins. Some women chose to undergo monitoring alone without ovarian stimulation, or self-monitoring at home with an LH-detection kit. The protocol for each patient was determined by their weight, previous medical history, and reason for infertility. Ovarian response was monitored by ultrasound follicle tracking combined with hormonal assessment (estradiol and/or luteinizing hormone). The cycle was cancelled if there was evidence of ovarian hyperstimulation. When at least one dominant follicle measured greater than 17 mm, ovulation was induced with hCG or recombinant LH.

### Sperm wash protocol

Semen was collected by masturbation into a sterile plastic specimen container either at home or in the clinic. Samples were processed within 15 min of arrival (no more than 60 min from the time of ejaculation). Semen was transferred into a 15 ml sterile tube containing a gradient consisting of 2 ml 45% and 2 ml 80% salinized silica (Gynotech; Malden, Netherlands) and centrifuged for 30 min at 400 g. The seminal plasma and gradient were aspirated and the remaining pellet was resuspended in 2 ml Sperm Wash Medium (Gynotech). Following a 6-min centrifugation at 300 g, the supernatant was removed, and the pellet was resuspended in 0.4 ml of Sperm Wash Medium.

### Insemination procedure

One insemination was performed approximately 36 h after administration of the ovulation triggering medication, employing a single use intra-uterine insemination cannula with shape memory (Laboratoire CCD, product code 12040MF), to allow angulation as required for intrauterine insemination. Women were instructed to take the ovulation triggering medication at 10:00 pm, and the IUI was performed 36 h after, around 10:00 a.m).

### Statistical analysis

Results were analyzed both by cycle and by couple (for a cumulative pregnancy rate). This cumulative pregnancy rate was calculated by grouping a patient’s cycles into a “round” of cycles; a round was finished when a β-HCG positive pregnancy was achieved, or treatment was stopped. A similar method is described by Lemmens, et al., 2016 [14].

In analyses completed per round, the mean NMSI over all cycles in a round was calculated and used to represent the overall NSMI in the round. To confirm that this was an appropriate representation, two Spearman Correlations were calculated. First, between mean NMSI per round and NMSI for each cycle, and second, between mean NSMI per round and the NMSI for the last cycle in the round. For each, a 95% confidence interval was calculated.

Results were summarized using descriptive statistics, as a count and percent of total, mean and standard deviation, or median and interquartile range. Results were summarized using the median and interquartile if the data was ordinal or not normally distributed as determined by the Shapiro-Wilk test. Clinical pregnancy rates and associated 95% confidence intervals were calculated. The Chi-Square test was used to compare the number of pregnancies achieved between the different OS protocols. The Chi-Square test for trend was used to compare the number of pregnancies achieved as female age, NMSI and number of mature follicles increased. Factors associated with the odds of achieving a clinical pregnancy were analyzed using binary logistic generalized estimating equations to control for clustering effects by couple. Patient factors including age, NMSI, duration of infertility, and cause of infertility were included, along with treatment factors such as number of follicles and ovulation induction protocol. *P* < 0.05 was considered statistically significant. Statistical analysis was completed with IBM SPSS Statistics v24.

## Results

A total of 737 couples and 2062 cycles were included in this study. The median female age at entry to care was 33.0 years [IQR 30–36.5]. Unexplained and female factor were the most common causes of infertility, and most patients had experienced infertility for greater than 1 year (Table 1).

**Table 1:** Baseline characteristics of the population by couple at entry to care

	Total Couples (*N* = 737)
Female Age (years), median [IQR]	33 (30–36.5)
Range	25–42
Female Age categories, n (%)	
< 30	151 (20.5)
30–34	305 (41.4)
35–39	234 (31.8)
≥ 40	46 (6.3)
Cause of Infertility, n (%)	
Male factor	138 (18.7)
Female factor	244 (33.1)
Male & female factor	49 (6.6)
Unexplained	274 (37.2)
Not recorded	32 (4.3)
Duration of infertility (months), n (%)	
< 12	64 (10.1)
12–23	285 (45.2)
24–35	156 (24.7)
≥ 36	126 (20.0)
Unknown, n	106

Characteristics of care are summarized in Table 2. The median number of total cycles per couple was 3 [IQR 2–4]. 85.6% of couples completed only one round of care; 11.8% completed two, and 2.6% completed ≥3 rounds. A total of 869 rounds of care were included, comprised of a median number of 2 cycles [IQR 1–3] per round. Among the 2062 cycles, the median NMSI per cycle was 22.4(× 10^6^) [IQR 7.8–53.2] and the median number of follicles per cycle was 2 [IQR 1–3].

**Table 2:** Characteristics of care

	Total Couples *N* = 737
Total Number of Rounds of Care Per Couple, n (%)	
1	631 (85.6)
2	87 (11.8)
3+	19 (2.6)
Total Cycles per Couple, median [IQR]	3 (2–4)
Range	1–22
Total Cycles per Couple, n (%)	
1	178 (24.2)
2	161 (21.8)
3	193 (26.2)
4	101 (13.7)
5–9	98 (13.3)
10+	6 (0.8)
	Total Rounds of Care *N* = 869
Number of Cycles per Round of Care, median [IQR]	2 [1–3]
Range	1–14
Total Number of Cycles per Round of Care, n (%)	
1	312 (35.9)
2	210 (24.2)
3	190 (21.9)
4	90 (10.4)
5–6	53 (6.1)
7–14	17 (1.5)
Total Cycles *N* = 2062	
NMSI per Cycle	
Median [IQR]	22.4 [7.8–53.2]
Unknown	3
Follicles per Cycle	
Median [IQR]	2 [1–3]
Unknown	141

In analyses by round, the average NMSI over all cycles was used. In order to examine the validity of using this summary measure we calculated the Spearman Correlation between mean NMSI per round and NMSI for all cycles (0.89; 95% CI 0.88–0.90). The Spearman Correlation between mean NMSI per round and NMSI for the last cycle in the round (0.93; 95% CI 0.92–0.94).

Pregnancy outcomes by cycles and by rounds are presented in Table 3. A total of 312 CPs resulted from 2062 cycles for an overall CP rate per cycle of 15.1% (95% CI 13.6–16.7). CP rates per cycle according to NMSI category, female age category, OS protocol, and number of mature follicles are also presented. The CP rate per round was 35.9% (95% CI 32.8–39.1). When divided into NMSI categories (× 10^6^) of < 1, 1–4, 5–9, and ≥ 10, the resulting CP rates were 8.9, 28.0, 35.1, and 38.7% per round, respectively (P Trend< 0.001). When divided into age categories of < 30, 30–34, 35–39, and ≥ 40 years, the resulting CP rates were 40.1, 37.4, 34.1, and 23.2% per round, respectively (P Trend = 0.03).

**Table 3:** Pregnancy outcomes by cycles and rounds

Total Cycles *N* = 2062	Total N (%)	Clinical Pregnancy N [Rate (95% CI)]	*P*-Value
Overall			
	2062 (100.0)	312 [15.1 (13.6–16.7)]^b^	
NMSI (×10^6^)			
< 1	80 (3.9)	3 [3.8 (1.1–9.7)]	0.0007^δ^
1–4	283 (13.7)	36 [12.7 (9.2–17.0)]	
5–9	245 (11.9)	30 [12.2 (8.6–16.8)]	
≥ 10	1454 (70.5)	243 [16.7 (14.9–18.7)]^b^	
Unknown	3		
Age (Years)			
< 30	348 (16.9)	59 [17.0 (13.3–21.2)]	0.09^δ^
30–34	862 (41.8)	135 [15.7 (13.4–18.2)]	
35–39	723 (35.1)	104 [14.4 (12.0–17.1)]	
≥ 40	129 (6.3)	14 [10.9 (6.4–17.1)]	
OS Protocol			
None	124 (6.1)	6 [4.8 (2.0–9.7)]	0.0003^β^
Letrozole or Clomid	210 (10.4)	22 [10.5 (6.9–15.2)]^a^	
Gonadotropins (± Letrozole or Clomid)	1690 (83.5)	280 [16.6 (14.9–18.4)]^a^	
Unknown	38		
Number of Mature Follicles			
1	805 (41.9)	101 [12.6 (10.4–15.0)]^a^	< 0.0001^δ^
2	565 (29.4)	86 [15.2 (12.4–18.4)]	
3	284 (14.8)	59 [20.8 (16.4–25.9)]^a^	
4	126 (6.6)	28 [22.2 (15.6–30.1)]	
≥ 5	141 (7.3)	29 [20.6 (14.5–27.8)]	
Unknown	141		
Total Rounds *N* = 869	Total N (%)	Clinical Pregnancy N [Rate (95% CI)]	
Overall			
	869 (100.0)	312 [35.9 (32.8–39.1)]	
Average NMSI (×10^6^)			
< 1	45 (5.3)	4 [8.9 (3.1–19.8)]	< 0.0001^δ^
1–4	93 (10.9)	26 [28.0 (19.6–37.6)]	
5–9	74 (8.7)	26 [35.1 (25.0–46.4)]	
≥ 10	643 (75.2)	249 [38.7 (35.0–42.5)]	
Unknown	14		
Age at Start of Round (Years)			
< 30	162 (18.6)	65 [40.1 (32.8–47.8)]	0.03^δ^
30–34	364 (41.9)	136 [37.4 (32.5–42.4)]	
35–39	287 (33.0)	98 [34.1 (28.8–39.8)]	
≥ 40	56 (6.4)	13 [23.2 (13.7–35.4)]	

^a^1 unknown clinical pregnancy outcome

^b^2 unknown clinical pregnancy outcomes

^δ^Χ^2^ test for trend

^β^Χ^2^ test

The impact of patient factors on the odds of clinical pregnancy per round of care are presented in Table 4. Increasing female age (OR = 0.96; 95% CI 0.92–0.99), average NMSI < 1.0 (× 10^6^) (OR = 0.21; 95% CI 0.07, 0.62) and duration of infertility ≥36 months (OR = 0.50, 95% CI 0.27–0.94) were associated with decreased odds of clinical pregnancy.

**Table 4:** Patient factors and odds of clinical pregnancy per round of care

*N* = 733	OR	95%CI	*P*-Value
Female Age			
Years	0.96	0.92–0.997	< 0.05
Average NMSI			
< 1	0.21	0.07–0.62	< 0.01
1–4	0.71	0.41–1.24	0.23
5–9	1.00	0.57–1.73	0.99
≥ 10	Ref	–	–
Duration of Infertility			
< 12	Ref	–	–
12–23	0.81	0.47–1.40	0.44
24–35	0.85	0.47–1.53	0.58
≥ 36	0.50	0.27, 0.94	< 0.05
Cause of Infertility			
Unexplained	1.19	0.84–1.69	0.34
Male & Female Factor	0.54	0.25–1.17	0.12
Male Factor	0.78	0.47–1.29	0.33
Female Factor	Ref	–	–

Table 5 presents two models of the impact of patient factors (Model 1) and patient and treatment factors (Model 2) on the odds of CP per cycle. In Model 1, CP odds were significantly decreased when NMSI was < 1 × 10^6^ (OR = 0.18; 95% CI 0.04–0.77) and when the etiology of infertility was male factor (OR = 0.64; 95% CI 0.41–0.99). In Model 2, CP odds were significantly decreased with NMSI < 1 × 10^6^ (OR = 0.20; 95% CI 0.05–0.91), male factor infertility (OR = 0.52; 95% CI 0.33–0.83), use of one mature follicle (OR = 0.59; 95% CI 0.39–0.89), and use of clomiphene or letrozole vs. gonadotropin or no OS treatment (OR = 0.51; 95% CI 0.31–0.85).

**Table 5:** Impact of patient factors (Model 1) and patient and treatment factors (Model 2) on the odds of Clinical pregnancy per cycle

	Model 1 *N* = 1795 Cycles from 626 Couples			Model 2 *N* = 1667 from 588 Couples		
	OR	95%CI	*P*-Value	OR	95%CI	*P*-Value
Female Age (Years)						
	0.97	0.94–1.01	0.11	0.97	0.93–1.00	0.056
NMSI						
< 1	0.18	0.04–0.77	0.021	0.20	0.05–0.91	0.036
1–4	0.87	0.55–1.40	0.57	0.81	0.49–1.34	0.42
5–9	0.77	0.48–1.24	0.29	0.77	0.47–1.27	0.31
≥ 10	Ref	–	–	Ref	–	–
Duration of Infertility (Months)						
< 12	Ref	–	–	Ref	–	–
12–23	0.86	0.52–1.42	0.56	0.98	0.59–1.65	0.95
24–35	0.99	0.58–1.67	0.96	0.99	0.57–1.70	0.97
≥ 36	0.60	0.34, 1.07	0.081	0.61	0.34–1.12	0.11
Cause of Infertility						
Unexplained	0.95	0.69–1.32	0.760.87	0.87	0.62–1.23	0.43
Male & Female Factor	0.52	0.19–1.46	0.21	0.58	0.21–1.59	0.29
Male Factor	0.64	0.41–0.99	0.045	0.52	0.33–0.83	0.006
Female Factor	Ref	–	–	Ref	–	–
Number of Follicles						
1				0.59	0.39–0.89	0.011
2				0.76	0.51–1.14	0.18
3				1.04	0.67–1.61	0.87
≥ 4				Ref	–	–
OS Drug Used						
None				0.52	0.18–1.50	0.22
Clomid or Letrozole				0.51	0.31–0.85	0.010
Gonadotropin				Ref	–	–

Model 1: adjusted for patient factors only

Model 2: adjusted for both patient and care-related factors

**Fig. 1:**
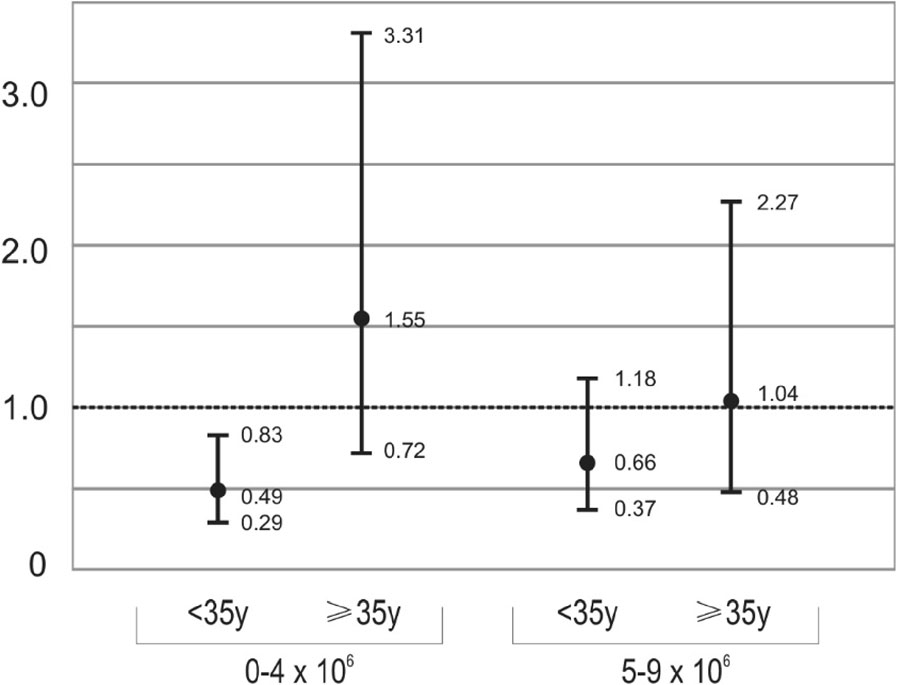
Odds of Clinical Pregnancy per cycle according to NMSI and Age Category

Models assessing the odds of CP per cycle according to female age category (< 35 vs. ≥35 years) are in Table 6. Due to small cell counts the < 1 and 1–4 NMSI categories were combined for these models. For females < 35 years, the odds of CP per cycle were reduced for NMSI < 5.0 vs. ≥10.0 (OR = 0.49; 95% CI 0.29–0.83); the odds of CP per cycle did not differ for NMSI 5.0–9.9 vs. ≥10.0. For those ≥35 years, no difference was seen for NMSI < 5.0 or 5.0–9.9 compared to ≥10.0. The adjusted model is similar (Fig. 1).

**Table 6:** Odds of Clinical Pregnancy per cycle according to NMSI and Age Category

Unadjusted Models		Model 1 Age < 35 (*N* = 1208 cycles from 455 couples)			Model 2 ≥35 (*N* = 852 cycles from 315 couples)	
NMSI Category	N	OR (95%CI)	*P*-Value	N	OR (95%CI)	*P*-Value
0–4	227	0.48 (0.30–0.77)	0.002	136	0.84 (0.46–1.54)	0.57
5–9	142	0.65 (0.38–1.11)	0.12	103	0.78 (0.39–1.54)	0.47
≥10	839	Ref	–	613	Ref	–
Adjusted Models		< 35 (*N* = 1053 cycles from 389 couples)			≥35 (*N* = 742 cycles from 269 couples)	
NMSI Category		OR (95%CI)	P-Value		OR (95%CI)	P-Value
0–4	204	0.49 (0.29–0.83)	0.007	119	1.55 (0.72–3.31)	0.26
5–9	127	0.66 (0.37–1.18)	0.17	94	1.04 (0.48–2.27)	0.92
≥10	722	Ref	–	529	Ref	–
Duration of Infertility (Months)						
< 12	76	Ref	–	97	Ref	–
12–23	501	0.82 (0.42–1.62)	0.57	354	0.91 (0.44–1.91)	0.81
24–35	268	0.98 (0.48–2.01)	0.96	138	0.91 (0.40–2.09)	0.82
≥ 36	208	0.51 (0.23–1.12)	0.09	153	0.70 (0.31, 1.59)	0.39
Cause of Infertility						
Unexplained	427	0.93 (0.62–1.38)	0.70	336	0.97 (0.60–1.65)	0.91
Male & Female Factor	51	0.53 (0.20–1.41)	0.21	65	0.45 (0.09–2.35)	0.35
Male Factor	218	0.93 (0.58–1.50)	0.77	138	0.21 (0.08–0.53)	0.001
Female Factor	357	Ref	–	203	Ref	–

Among the 312 recorded clinical pregnancies, outcomes on 262 pregnancies were available (Table 7). The outcomes of 50 CPs were unknown, as antenatal and delivery care was not completed at our center. Among those 262 pregnancies, there were 220 live births, 36 losses, and 6 who were still pregnant at the time of chart review. Detailed chart reviews were conducted on the 196 participants who delivered at our center. Among these patients, 79.1% delivered at term, 59.7% delivered vaginally, and 82.7% had a singleton delivery. In terms of pregnancy complications, 10.7% experienced a hypertensive disorder and 8.7% had gestational diabetes. Among the term babies, the average birthweight was 3404 ± 542 g and 97.1% had a normal APGAR score (≥7) at 5 min.

**Table 7:** Live birth outcomes

Outcomes for all Positive Fetal Heart Rate, n (%)	*N* = 312
Live Births	220 (84.0)
Miscarriages^a^	36 (13.7)
Not Yet Delivered	6 (2.3)
Unknown, n	50
Pregnancy Outcomes for all deliveries at Kingston General Hospital	*N* = 196
GA at delivery (weeks), n (%)	
Extremely preterm (< 28)	5 (2.6)
Very preterm (28–31)	3 (1.5)
Moderate preterm (32–33)	3 (1.5)
Late preterm (34–37)	30 (15.3)
Term (≥37)	155 (79.1)
Type of Delivery, n (%)	
Spontaneous Vaginal	97 (49.5)
Assisted Vaginal	20 (10.2)
Cesarean Section	79 (40.3)
Number of Babies, n (%)	
Singleton	162 (82.7)
Twins	30 (15.3)
Triplets	4 (2.0)
Pregnancy Complications, n (%)	
Gestational Diabetes	17 (8.7)
Hypertensive Disorder	21 (10.7)
Pre-existing Hypertension	3 (1.5)
Outcomes for all babies delivered at Kingston General Hospital (≥37 weeks gestation)	*N* = 172
Birthweight (grams), mean (SD)	3404 (542)
< 2500, n (%)	10 (5.9)
2500–2999, n (%)	27 (15.9)
3000–3999, n (%)	110 (64.7)
≥4000, n (%)	23 (13.5)
Unknown, n	2
APGAR Scores, n (%)	
Normal (≥7) at 1 min	151 (87.8)
Normal (≥7) at 5 min	167 (97.1)
Unknown, n	3
Outcomes for all babies delivered at Kingston General Hospital	*N* = 236
APGAR Scores, n (%)	
Normal (≥7) at 1 min	184 (78.0)
Normal (≥7) at 5 min	213 (90.3)
Unknown, n	13

^a^includes elective terminations

## Discussion

In this study, 312 CPs were achieved from 2062 IUI cycles, resulting in a 15.1% CP rate per cycle and 35.9% per round. These pregnancy rates are comparable to those of other reports [7, 9, 10, 12]. During statistical analysis in studies such as this, it is important to consider that multiple cycles in the same patient are not independent of one another, and this may influence results. This is not taken into consideration in per cycle results, but for this reason, we also analyzed the data according to rounds of care. Other strengths of this study include the large cohort of patients, the heterogeneous indications, and wide age range of patients (including 129 cycles in patients over 40 years of age).

There is discrepancy in the primary outcomes used in many studies on IUI outcomes, as some use serum-positive pregnancy rate [7], and others use the presence of a gestational sac and fetal heartbeat [10, 13, 15] or delivery rate [8]. We present results for the latter two outcomes, recognizing that data about live births was available only for 83% of the clinical pregnancies.

Recommended minimum NMSI thresholds for IUI vary widely across the literature, with reports of 1 million [8], 2 million [7], 5 million [10], and 10 million [9], when calculated for female patients of all ages. It is important to elicit the impact of differing NMSI levels that may exist according to female age, which is known to be an independent predictor of success following IUI [11].

When not stratified by age category, our results showed that the odds of CP per round were significantly decreased with increasing female age, average NMSI < 1.0 (× 10^6^), and duration of fertility ≥36 months. Odds of CP per cycle were likewise significantly reduced when the NMSI was < 1.0, and with increasing female age when adjusted for both patient- and care-related factors.

Only a few studies have considered NMSI according to female age; Demir et al. found that pregnancy rates were only significantly different in the group of women < 25 years when NMSI was > 10 × 10^6^, compared to age groups 25–30 and > 30, and NMSI categories of < 5 and 5–10 [13]. Similarly, Badawy, et al. found that pregnancy rates were significantly different also in the group of women < 25 years, but only when NMSI was > 5 × 10^6^ (compared to any NMSI category < 5 and women 25–30, 30–35, and 35–40 years) [12]. Both of these results argue against the NMSI as a useful predictor of success in patients above the age of 25 [12, 13].

In our study, pregnancy rates were only significantly different in the group < 35 years when NMSI was < 5.0, suggesting that NMSI is not a good predictor of success in patients over 35 years. Although this study only considered two categories of ages (< 35 and ≥ 35), our finding is consistent with those of the aforementioned studies, in that the NMSI is not a useful predictor of success in older patients [12, 13].

When compared in groups < 35 and ≥ 35 years, odds of CP per cycle were significantly reduced only in patients < 35 years and NMSI 0–4, when either adjusted or unadjusted for other patient-related factors. This unexpected result of lower pregnancy rates in patients < 35 vs. those ≥35 within the same NMSI category may be due to selection bias. It is possible that patients who presented at increased maternal age with perceived unfavourable characteristics for IUI were referred to other ARTs earlier or did not proceed with IUI at all. Likewise, those patients presenting with low NMSI initially may have been referred earlier, resulting in the relatively small cohort of patients with NMSI < 1 × 10^6^ in this study.

The NMSI may have unique value as a prognostic tool in that it reflects both sperm concentration and motility, as well as the effects of sperm processing [6]. The limitation is that it cannot be used for counselling during the initial infertility workup, but only during/after the IUI procedure. As such, the utility of the NMSI as predictor of pregnancy rates has been questioned. The baseline TMSC and sperm morphology will guide the pregnancy rates counselling during the initial infertility workup. If with the baseline sperm parameters a couple is eligible for IUI, the NMSI will help to determine during the course of IUI if a couple is not suitable anymore for this type of treatment and should move to different ART options.

## Conclusions

These results suggest that NMSI can be used as a predictor of success in IUI in patients who are < 35 years of age; these patients may be advised to pursue other ARTs when NMSI is < 5.0 × 10^6^. In patients ≥35 years, NMSI does not appear to be a useful predictor of success; more research is needed to determine other factors that are predictive of success with IUI in this age group.

## Data Availability

The datasets analysed during the current study are available from the corresponding author on reasonable request.

## Abbreviations

ART: Assisted reproductive technologies
CP: Clinical pregnancy
ICSI: Intracytoplasmic sperm injection
IUI: Intrauterine Insemination
IVF: In vitro fertilization
NMSI: Motile spermatozoa inseminated
OS: Ovarian stimulation
